# Correlation Between Electrode Location and Clinical Efficacy of Deep Brain Stimulation of the Subthalamic Nucleus in Isolated Generalized Dystonia

**DOI:** 10.3390/jcm15093346

**Published:** 2026-04-28

**Authors:** Jingchao Wu, Guanyu Zhu, Jianguo Zhang

**Affiliations:** 1 Department of Neurosurgery, Tianjin Huanhu Hospital, Tianjin 300222, China; 2 Tianjin Key Laboratory of Cerebral Blood Flow Reconstruction and Head and Neck Tumor New Technology Translation, Tianjin 300222, China; 3 Department of Neurosurgery, Beijing Tiantan Hospital, Capital Medical University, Beijing 100070, China

**Keywords:** dystonia, STN, DBS, electrode location, isolated generalized dystonia

## Abstract

**Background**: Isolated Generalized Dystonia (IGD) severely reduces quality of life. Deep brain stimulation of the subthalamic nucleus (STN-DBS) is effective for refractory IGD, but the factors influencing efficacy remain unclear. **Methods**: Twelve IGD patients treated with bilateral STN-DBS (2016–2021) were retrospectively analyzed. Clinical outcomes (BFMDRS, HAMA, HAMD, MOCA, MMSE) were evaluated at baseline and the last follow-up (12–60 months). The electrode position and volume of tissue activated (VTA) in STN subregions were analyzed using Lead-DBS V3.0. **Results**: STN-DBS significantly improved BFMDRS-M and BFMDRS-D scores (*p* < 0.001) without cognitive or mood deterioration. BFMDRS-M improvement correlated positively with the VTA of the whole STN and motor subregion (*p* < 0.05) but not with associative/limbic subregions. The preoperative HAMD score was negatively associated with motor improvement (*p* = 0.002). **Conclusions**: STN-DBS safely improves motor function in IGD. Efficacy depends on the VTA within the STN sensorimotor subregion. The preoperative HAMD score may predict the long-term outcome, helping guide patient selection and surgical planning.

## 1. Introduction

Dystonia is a movement disorder characterized primarily by abnormal movements or postures caused by sustained or intermittent muscle contractions [1]. Isolated Generalized Dystonia (IGD) must simultaneously meet the diagnostic criteria for both isolated dystonia and generalized dystonia. This condition severely impacts patients’ quality of life and poses growing clinical challenges. Deep brain stimulation (DBS) is an important treatment method for refractory dystonia [2,3]. In most clinical studies, the severity of dystonia in patients with primary dystonia generally improved by 50–60% [4,5,6]. However, the results are often variable, with randomized controlled trial results showing up to 25% non-responders (improvement rate < 25%), even in carefully selected groups of primary dystonia patients [4].

Our previous study confirmed that both the globus pallidus interna (GPi) and the subthalamic nucleus (STN), as DBS targets, reduce BFMDRS motor and disability scores and improve quality of life in IGD patients [7]. However, the factors influencing the efficacy of DBS in the treatment of IGD remain poorly understood at present. Furthermore, the delayed improvement of dystonic symptoms after DBS activation complicates the programming process [8]. In Meige syndrome, the volume of activated tissue in the subthalamic nucleus (STN) is positively correlated with the improvement of motor symptoms following DBS surgery [9]. To date, recent advances in computational neuroscience have established computer-assisted modeling and simulation as essential tools for optimizing DBS targeting, electrode localization, and volume of tissue activated (VTA) calculation [10]. These techniques enable precise quantification of stimulation effects within deep brain nuclei and their functional subregions, improving the understanding of structure–efficacy relationships. For isolated generalized dystonia (IGD), only one previous study [11] explored the correlation between STN VTA and clinical improvement, with a very small sample (*n* = 5) and no analysis of STN functional subdivisions. Therefore, the specific structural basis underlying STN-DBS efficacy remains unclear. This study aims to investigate the correlation between electrode location, VTA, and clinical outcomes in IGD patients receiving DBS in the STN. Additionally, we incorporate disease duration, age at surgery, age at onset, baseline BFMDRS-M scores, and scores from HAMA, HAMD, MMSE, and MOCA into a linear regression model to aid in patient selection and outcome prediction.

## 2. Materials and Methods

### 2.1. Patients

This study included patients with IGD who underwent bilateral STN-DBS treatment in the Department of Functional Neurosurgery, Beijing Tiantan Hospital, Capital Medical University, from January 2016 to December 2021, and had complete follow-up data. The follow-up duration ranged from 12 to 60 months. Inclusion criteria: (1) Diagnosis of IGD confirmed by movement disorder specialists; (2) preoperative MRI showing no structural abnormalities (e.g., tumors, hemorrhage); (3) no history of severe psychiatric/cognitive disorders, epilepsy, thalamotomy, or other neurosurgical procedures affecting neural function; (4) age ≥ 16 years at the time of cognitive/psychological assessment. Exclusion criteria: (1) Secondary dystonia (e.g., post-stroke, neurodegenerative disease); (2) MRI evidence of brain structural lesions; (3) patients with MOCA < 10 were strictly excluded from the study to avoid enrollment of individuals with severe cognitive impairment, ensuring the reliability of cognitive and psychological assessments. A total of 12 IGD patients were enrolled. This study was conducted in accordance with the Declaration of Helsinki and was approved by the Ethics Committee of Beijing Tiantan Hospital (protocol code: HX-A-2021006, ethics approval number: KY2022-006-02, approval date: 6 April 2022). Written informed consent was obtained from all the patients or their guardians.

### 2.2. Surgical and Programming Procedures

All the patients underwent 3.0 T magnetic resonance imaging (MRI; MAGNETOM Prisma, Siemens, Erlangen, Germany) 1 day preoperatively, including 3d T1 and T2 navigation scans for high-resolution brain structural imaging to support target localization and surgical planning. Surgery was performed under general anesthesia (GA). Bilateral arc incisions were made on the forehead; after hemostasis, skull drilling and dural cauterization were conducted. Guided by the stereotactic frame, catheters and recording electrodes were inserted, and microelectrode recording (MER) confirmed STN localization before implanting deep brain electrodes (PINS L301, PINS Medical Co., Beijing, China; Medtronic 3389, Medtronic Inc., Minneapolis, MN, USA). Postoperatively, a thin-slice head CT was performed to rule out complications (e.g., intracranial hemorrhage). SurgiPlan fused the preoperative MRI and postoperative CT to verify electrode position deviation. After 3–7 days of external stimulation to exclude acute adverse effects (e.g., dysarthria, paresthesia, muscle spasm) and confirm safe stimulation parameters, a second-stage IPG (implantable pulse generator) implantation was performed under GA (PINS G102/G102RZ, PINS Medical Co., Beijing, China; or Activa RC, Medtronic Inc., Minneapolis, MN, USA), with the IPG placed under the clavicle.

### 2.3. Clinical Assessments

All the patients underwent standardized video recordings for dystonia evaluation. Assessments were performed preoperatively and at the last follow-up (12–60 months post-DBS activation). The Burke–Fahn–Marsden Dystonia Rating Scale (BFMDRS) was used: BFMDRS-M (motor subscale, 0–120 points) evaluates dystonia severity across body regions, and BFMDRS-D (disability subscale, 0–30 points) assesses functional impairment. Hamilton Anxiety Scale (HAMA): A 14-item scale (0–4 points per item) evaluating anxiety severity. Hamilton Depression Scale (HAMD): A 24-item scale (0–4 points per item) evaluating depression severity. Montreal Cognitive Assessment (MOCA): A 30-item scale assessing multiple cognitive domains. Mini-Mental State Examination (MMSE): A 30-item scale for rapid cognitive screening.

### 2.4. DBS Electrode Localization and VTA Calculation

Lead-DBS V3.0 software (https://www.lead-dbs.org/) was used for electrode localization, with coordinates converted to MNI and AC-PC standard spaces. VTA was calculated using Lead-DBS V3.0 based on stimulation parameters at the last follow-up (frequency, pulse width, voltage, stimulation mode). Overlap analysis: VTA overlap with STN was quantified (mm^3^).

### 2.5. Statistical Analysis

All the analyses were performed using SPSS 23.0 (IBM Corp., Armonk, NY, USA). Continuous variables were presented as mean ± standard deviation (SD). Paired *t*-tests or Wilcoxon signed-rank tests were used for preoperative–postoperative comparisons. Pearson correlation analysis was used to assess the relationship between the BFMDRS improvement rate and VTA (correlation coefficient R: >0.5 = strong, 0.3–0.5 = weak, <0.3 = no correlation).

Clinical variables (disease duration, age at surgery/onset, baseline BFMDRS-M, HAMA, HAMD, MMSE, MOCA) were included in univariate linear regression models. Statistical significance was set at *p* < 0.05.

## 3. Results

### 3.1. Clinical Data

A total of 12 patients underwent STN-DBS (8 males, 4 females). The mean age at surgery was 19.67 ± 16.67 years, mean age at onset was 14.08 ± 17.89 years, mean disease duration was 65 ± 87.18 months, and mean follow-up duration was 12–60 months. Stimulation parameters at last follow-up are shown in Table 1.

### 3.2. Clinical Efficacy of STN-DBS

**Figure 1:**
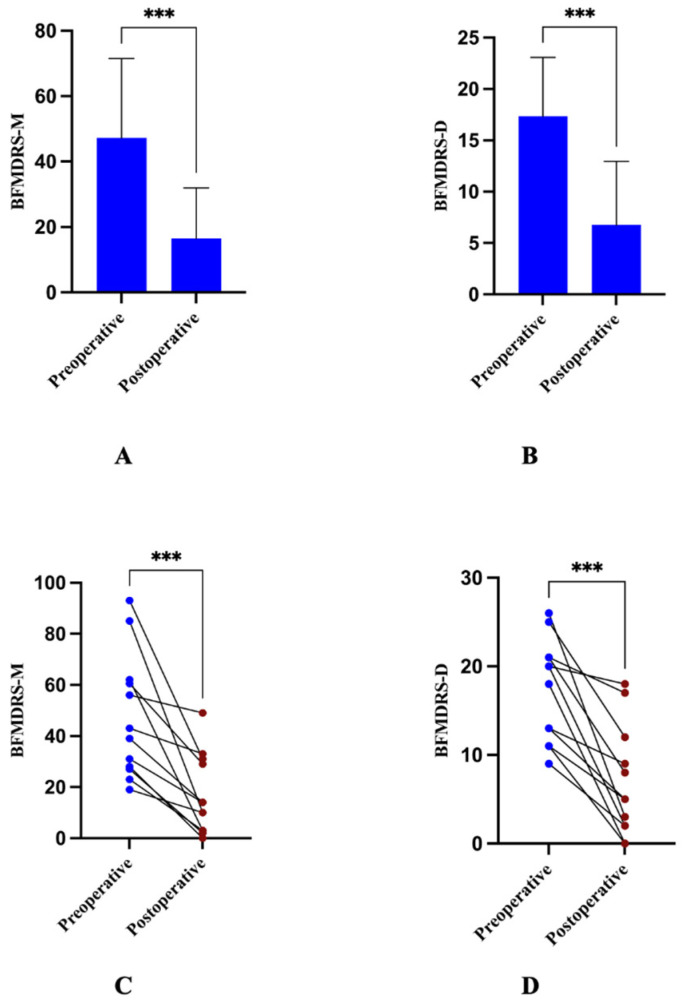
Dystonia outcomes after STN-DBS. (**A**,**B**): Postoperative changes in dystonia severity as measured by the BFMDRS-M (**A**) and BFMDRS-D (**B**). (**C**,**D**): Line plots showing individual patient baseline and last follow-up BFMDRS-M (**C**), BFMDRS-D (**D**). Higher values indicate more severe dystonia. *** *p* < 0.001 by two-tailed paired-samples *t*-test.

**Figure 2:**
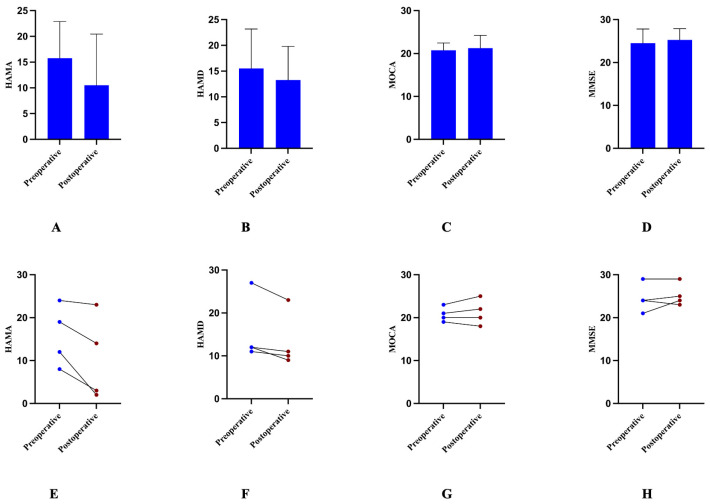
Mood and global cognition outcomes. (**A**,**B**): Postoperative changes in mood were examined by HAMA (**A**) and HAMD (**B**). (**C**,**D**): Postoperative changes in global cognition were examined by MOCA (**C**) and MMSE (**D**). Line plots showing individual patient baseline and last follow-up HAMA (**E**), HAMD (**F**), MOCA (**G**) and MMSE (**H**). Higher values indicate more severe dystonia.

The postoperative BFMDRS-M and BFMDRS-D scores were significantly lower than preoperative scores. The mean BFMDRS-M score decreased from 47.21 ± 24.32 preoperatively to 16.50 ± 15.49 at last follow-up (*p* = 0.0006). The mean BFMDRS-D score decreased from 17.33 ± 5.74 preoperatively to 6.75 ± 6.20 at last follow-up (*p* = 0.0002). Individual patient changes in BFMDRS scores are shown in Figure 1. There were no significant improvements in the HAMA or HAMD scores. The mean HAMA score decreased from 15.75 ± 7.14 preoperatively to 10.50 ± 9.95 at last follow-up (*p* = 0.0652). The mean HAMD score decreased from 15.50 ± 7.68 preoperatively to 13.25 ± 6.55 at last follow-up (*p* = 0.0577). The MOCA and MMSE scores showed no significant changes. The mean MOCA score increased from 20.75 ± 1.71 preoperatively to 21.25 ± 2.99 at last follow-up (*p* = 0.4950). The mean MMSE score increased slightly from 24.50 ± 3.32 preoperatively to 25.25 ± 2.63 at last follow-up (*p* = 0.4444) (Figure 2).

### 3.3. The Active Contact Locations in MNI/AC-PC and Its Relationship with the Improvement Rate of Motor Symptoms in IGD Patients

MNI Space: The mean coordinates of left-sided activation contacts were X = −12.26 ± 0.72 mm, Y = −12.96 ± 0.76 mm, Z = −6.30 ± 1.36 mm. The mean coordinates of right-sided contacts were X = 13.05 ± 0.66 mm, Y = −12.30 ± 0.96 mm, Z = −6.07 ± 1.16 mm. AC-PC Space: The mean coordinates of left-sided activation contacts were X = −11.97 ± 0.73 mm, Y = −1.30 ± 0.85 mm, Z = −2.21 ± 1.15 mm. The mean coordinates of right-sided contacts were X = 12.73 ± 0.54 mm, Y = −0.48 ± 0.78 mm, Z = −2.40 ± 1.10 mm. According to the improvement rate of the Burke–Fahn–Marsden Dystonia Rating Scale-Motor (BFMDRS-M), patients were divided into responders (improvement rate > 50%), intermediate responders (improvement rate 25–50%), and non-responders (improvement rate < 25%). Active contact coordinates in MNI and AC-PC spaces are presented descriptively across responder subgroups (Table 2 and Table 3). Formal statistical comparison was not performed due to the highly unbalanced and small subgroup sizes (*n* = 2, *n* = 1, *n* = 9), which preclude reliable statistical inference.

### 3.4. Relationship Between STN/Subregion VTA and BFMDRS Improvement Rate

**Figure 3:**
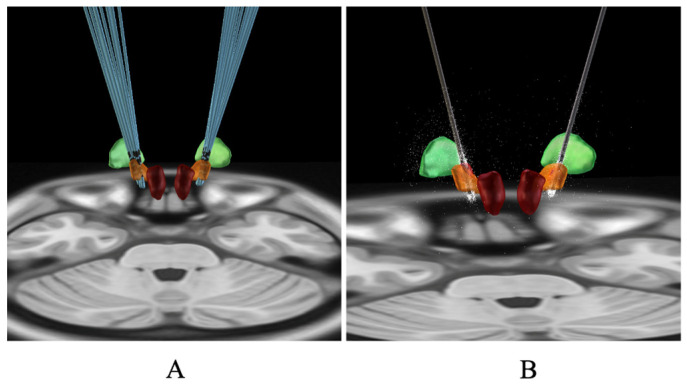
3D illustration of all active electrode contacts: (**A**) Electrode position of 12 IGD patients. (**B**) Relationship between volume of tissue activated (VTA) in STN. Yellow nucleus: STN. Red nucleus: red nucleus. Green nucleus: GPi.

**Figure 4:**
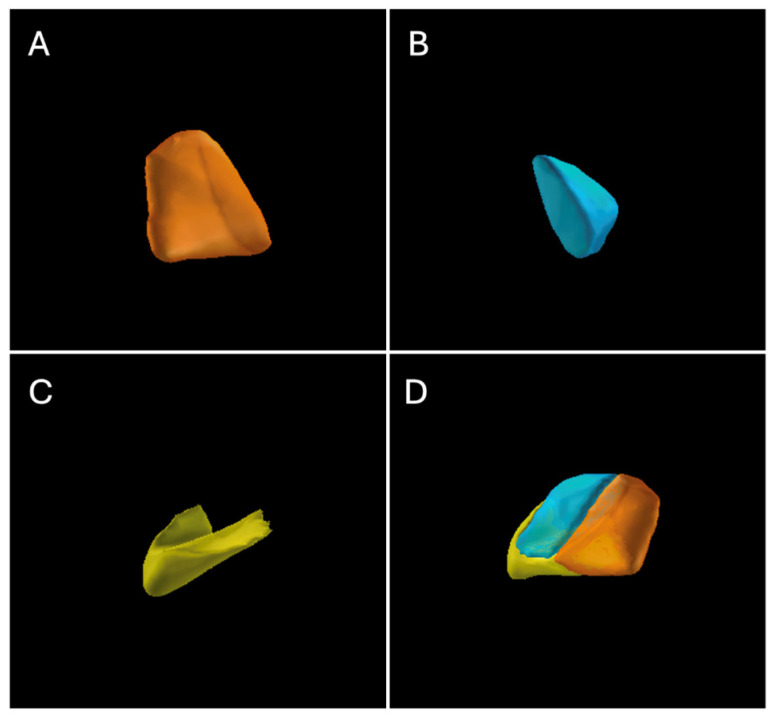
STN and subregions: (**A**) STN Sensorimotor; (**B**) STN Associative; (**C**) STN Limbic; (**D**) STN.

The STN was subdivided into three subregions: sensorimotor subregion, associative subregion, and limbic subregion (Figure 3 and Figure 4). The mean VTA volumes of STN subregions were motor (37.44 ± 16.04 mm^3^), associative (34.09 ± 23.64 mm^3^), limbic (18.77 ± 34.79 mm^3^), and total STN (70.56 ± 39.84 mm^3^). At the last follow-up, compared with the baseline score before surgery, the improvement rate of the BFMDRS-M score after surgery showed a significant positive correlation with the VTA of the STN and motor subregion (*p* = 0.005, *p* = 0.04; Table 4) but no significant correlation with the VTA of associative and limbic subregion (*p* > 0.05; Table 4).

### 3.5. Predictors of Motor Outcomes

**Figure 5:**
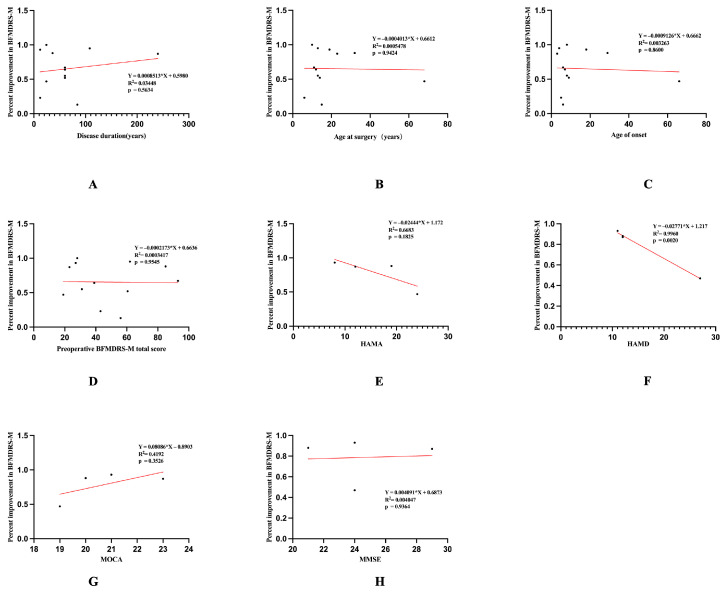
Predictors of motor outcomes after STN-DBS. A simple linear regression model was constructed to assess candidate predictive factors for movement outcomes, including baseline variables (demographic and clinical). Significant negative correlations were observed between motor outcome and preoperative HAMA (**E**) and preoperative HAMD (**F**), while Disease duration (**A**), Age at surgery (**B**), Age of onset (**C**), Preoperative BFMDRS-M total score (**D**), MMSE score (**H**) and MOCA score (**G**) showed no significant correlations.

Univariate linear regression included the following variables: disease duration, age at surgery, age at onset, preoperative BFMDRS-M, HAMA, HAMD, MMSE, and MOCA. In the numerical variables, HAMD was negatively correlated with the improvement rate of BFMDRS-M (*p* = 0.002). There was no significant correlation between the improvement rate of BFMDRS-M and disease duration, age at surgery, age of onset, baseline BFMDRS-M score, HAMA, MMSE, or MOCA (Figure 5).

## 4. Discussion

DBS targeting STN has evolved into a promising therapeutic option for isolated dystonia, complementing GPi-DBS. Numerous studies have compared the therapeutic efficacy of STN-DBS and GPi-DBS for the treatment of isolated dystonia, yet a definitive conclusion has not been reached due to inconsistent findings across these investigations [12,13,14]. STN and its adjacent white matter regions may potentiate a more robust output from the basal ganglia to the cortical areas. This enhancement of neural signal transmission contributes to the amelioration of symptoms in patients with dystonia [15]. This study provides the 12- to 60-month follow-up results of STN-DBS in the treatment of IGD. The results show that STN-DBS significantly improved the scores of the BFMDRS-M and BFMDRS-D.

Our study found that there were no significant changes in MMSE and MOCA scores in patients with isolated dystonia before and after STN-DBS surgery, which was generally consistent with the findings of previous studies [16]. This result indicates that the global cognitive function remains stable in dystonia patients following STN-DBS treatment. In a stark contrast, cognitive impairments, particularly in verbal fluency and executive control, have been reported in patients with Parkinson’s disease (PD) after STN-DBS [17]. One proposed mechanism underlying the STN-DBS-induced cognitive decline in PD patients is the disruption of the hyperdirect pathway caused by high-frequency stimulation (>100 Hz) [18]. As a critical neural network connecting STN with other basal ganglia structures and relevant cortical regions, the hyperdirect pathway is involved in motor control and certain cognitive functions. Interference with the normal function of this pathway by high-frequency stimulation may consequently lead to cognitive decline in PD patients. However, the absence of significant cognitive impairment in dystonia patients suggests distinct pathophysiological mechanisms and etiologies between dystonia and PD. For instance, the progressive neurodegeneration and dopamine depletion in the striatal and cortical dopamine networks, which are characteristic of PD, ultimately result in executive dysfunction and increased susceptibility to stimulation-induced cognitive decline [19]. Given the regulatory role of the STN in both motor and cognitive pathways in PD, high-frequency DBS stimulation of this region exerts a more pronounced cognitive impact. In contrast, the pathophysiological mechanisms of dystonia may preserve the utilization of cognitive reserve even with STN stimulation. This may be attributed to the fact that dystonia primarily involves the neural mechanisms underlying motor control, with minimal involvement of the neural networks mediating cognitive functions [20]. Unlike PD patients, dystonia patients do not exhibit extensive degeneration or depletion in the cognition-related dopamine pathways, despite abnormal motor control [16]. Therefore, these patients can still utilize cerebral cognitive reserve to maintain global cognitive function after STN-DBS treatment.

Our study demonstrates a significant positive correlation between the VTA within STN and BFMDRS-M scores at long-term follow-up. Previous studies have shown that the STN can be functionally subdivided into the sensorimotor region, associative region, and limbic region [21]. Among these, the sensorimotor region of the STN is closely associated with beta-band oscillations and shows clear anatomical connections with the motor cortex [22,23]. This suggests that this region not only plays a critical role in motor control but may also influence the manifestation of movement disorders during pathological processes. To the best of our knowledge, our study is the first to investigate the impact of VTAs in different STN subregions on the surgical outcomes of IGD. Through comparative analysis, we verified the clinical efficacy of STN-DBS and found that only VTA located within the STN and those within the STN sensorimotor subregion were strongly associated with postoperative prognosis in IGD patients. Therefore, our findings expand the current understanding of STN-DBS and may ultimately improve the treatment of IGD. In addition to validating clinical efficacy, our study deepens insights into the mechanisms underlying STN-DBS therapy and provides novel perspectives for the future treatment of IGD. Notably, more precise target localization and individualized therapeutic strategies may enhance treatment outcomes for IGD patients. These findings lay a foundation for further exploring the specific role and mechanisms of VTA in STN-DBS. Collectively, our study not only provides new scientific evidence supporting the application of STN-DBS in IGD patients but also promotes further advances in this field, ultimately aiming to improve treatment outcomes and quality of life for affected individuals.

To date, there is no general consensus on the predictive factors for the clinical efficacy of STN-DBS in the treatment of isolated dystonia [24,25]. Conclusions from different studies are inconsistent, posing challenges for clinicians in formulating treatment plans. Dystonia is generally classified into primary and secondary types. Primary dystonia is caused by specific genetic factors or mutations, usually presenting with single symptoms and favorable prognosis; patients with primary dystonia often show a significantly more positive response to DBS. Some studies have demonstrated that patients with primary dystonia can achieve more than 50% improvement in symptoms after DBS treatment [26,27]. In contrast, patients with secondary dystonia (e.g., caused by brain injury, drug intoxication, or other neurological diseases) are often accompanied by other neurofunctional impairments, and the therapeutic effect of DBS is usually poor because electrical stimulation is insufficient to reverse multiple neuronal damages caused by complex pathological processes. Patient age and disease duration are widely recognized as important factors affecting surgical outcomes [28,29]. This phenomenon can be attributed to the fact that the neural function of young patients is still in the developmental stage, with better adaptability and repair capacity [30]. However, conflicting research results have emerged. For example, Xu et al. [31] found no significant correlation between clinical outcomes and disease duration or surgical age, suggesting that we should not consider these factors unilaterally but conduct in-depth case-by-case analysis. We found a negative correlation between preoperative HAMD scores and long-term clinical improvement. Due to the small number of cases considered, our results may be biased. The inconsistency in predictive factors identified by different studies may be attributed to the limited number of cases and differences in the methods adopted. Given the preliminary nature of this finding and the small sample size, this result should be interpreted with caution. It raises the hypothesis that preoperative depressive symptoms might be associated with less favorable motor outcomes and suggests that further research is warranted to explore whether psychological or psychiatric interventions could potentially optimize DBS outcomes in patients with isolated generalized dystonia. At this stage, no definitive clinical recommendation can be made.

To better evaluate and optimize the efficacy of STN-DBS in the treatment of dystonia, future studies may focus on the following directions: (1) Expand sample size: Conduct multi-center collaborative studies to collect data with a larger sample size, so as to enhance the credibility and generalizability of the results. (2) Conduct prospective studies: Establish long-term longitudinal observation studies to track the postoperative recovery of patients and analyze the key factors affecting therapeutic outcomes. (3) Establish unified standards: Develop standardized criteria for case inclusion and evaluation indicators to improve the comparability between results of different studies. (4) Consider multi-dimensional factors: Focus not only on biological factors but also combine the patient’s psychosocial background to conduct multi-dimensional comprehensive analysis and establish a comprehensive predictive model.

## 5. Conclusions

Our study demonstrates that STN-DBS effectively improves motor function in IGD patients without significant cognitive impairment, with therapeutic efficacy primarily mediated by VTA in the STN sensorimotor subregion. Pre-operative HAMD scores may serve as a potential predictor of long-term outcomes. These findings enhance our understanding of STN-DBS mechanisms and provide practical guidance for optimizing surgical and post-operative management. Addressing the unresolved issues and pursuing the proposed future directions will further advance the application of STN-DBS, ultimately improving the quality of life for IGD patients.

## Figures and Tables

**Table 1:** Characteristics and stimulation parameters of each patient with generalized isolated dystonia.

Case	Sex	Age at Onset (yrs)	Age at Surgery (yrs)	Duration of Symptoms (mos)	Gene Mutation	Stimulation Parameters with Best Response (Contact, Pulse Width, Frequency, Amplitude, Left/Right)
1	M	8	13	60	KMT2B	Case(+)7(−)/Case(+)2(−), 80/60, 140/140, 2.1/2.0
2	M	18	19	12	NA	Case(+)10(−)/Case(+)2(−), 60/60, 130/130, 1.9/2.0
3	M	66	68	24	No	Case(+)6(−)/Case(+)3(−), 80/70, 150/150, 2/2.25
4	F	29	32	36	No	Case(+)5(−)/Case(+)2(−)3(−), 80/80, 130/130, 2.5/2.4
5	F	7	12	60	NA	Case(+)6(−)/Case(+)2(−), 90/80, 145/145, 2.45/2.25
6	M	4	13	108	No	Case(+)6(−)/Case(+)2(−), 60/60, 130/130, 2.2/2.2
7	M	8	10	24	TOR1A	Case(+)5(-)8(−)/Case(+)2(−)4(−), 60/60, 130/130, 2.5/1.7
8	F	5	6	12	ANO3	Case(+)9(−)/Case(+)1(−), 60/60, 110/110, 1.8/1.9
9	M	3	23	240	No	Case(+)10(−)/Case(+)2(−), 60/60, 130/130, 1.9/1.8
10	F	6	15	84	No	Case(+)5(−)6(−)/Case(+)1(−)2(−), 60/70, 130/130, 1.65/1.75
11	M	9	14	60	No	Case(+)8(−)/Case(+)4(−), 70/60, 140/140, 2.4/1.5
12	M	6	11	60	No	Case(+)7(−)/Case(+)3(−), 60/60, 130/130, 1.9/2.1

F, female; M, male; none, no gene mutation; NA, not available (patient refused genetic testing).

**Table 2:** Comparison results of active contact coordinates in MNI space in different BFMDRS-M Improvement rate groups [M (IQR), mm].

Group	Number of Cases	Left			Right		
		X	Y	Z	X	Y	Z
Improvement rates < 25% group	2	−11.90 ± 0.16	13.13 ± 0.28	−7.06 ± 0.17	12.68 ± 0.63	−12.66 ± 1.27	−6.95 ± 0.51
Improvement rates 25–50% group	1	−13.73	−13.66	−6.71	13.95	−12.75	−4.52
Improvement rates > 50% group	9	−12.17 ± 0.63	−12.84 ± 0.83	−6.09 ± 1.53	13.03 ± 0.65	−12.17 ± 0.99	−6.05 ± 1.15

**Table 3:** Comparison results of active contact coordinates in AC-PC space in different BFMDRS-M Improvement rate groups [M (IQR), mm].

Group	Number of Cases	Left			Right		
		X	Y	Z	X	Y	Z
Improvement rates < 25% group	2	−12.74 ± 0.80	−1.62 ± 0.38	−2.33 ± 0.80	13.27 ± 0.99	−0.70 ± 0.23	−2.99 ± 0.06
Improvement rates 25–50% group	1	−12.25	−2.62	−2.32	12.35	−0.07	−0.90
Improvement rates > 50% group	9	−11.76 ± 0.67	−1.08 ± 0.83	−2.17 ± 1.32	12.65 ± 0.54	−0.48 ± 0.89	−2.44 ± 1.14

**Table 4:** The correlation between VTA and the BFMDRS improvement rate.

	BFMDRS-M Improvement Rate	
	R	*p*
STN Sensorimotor	0.597	0.04
STN Associative	−0.059	0.854
STN Limbic	−0.469	0.124
STN	0.751	0.005

STN = STN Sensorimotor + STN Associative + STN Limbic; R, r value; *p* < 0.05.

## Data Availability

The data presented in this study are available on request from the corresponding author. The data are not publicly available due to privacy and ethical restrictions.
